# Sacral nerve stimulation lead implantation using the o-arm

**DOI:** 10.1186/1471-2490-13-48

**Published:** 2013-10-16

**Authors:** Pekka A Hellström, Jani Katisko, Pertti Finnilä, Markku H Vaarala

**Affiliations:** 1 Division of Operative Care and Medical Research Center, Oulu University Hospital, P.O. Box 20, Oulu, FIN 90029 OYS, Finland

**Keywords:** Transcutaneous electric nerve stimulation, Prostheses and implants, Bladder, Colon, Image-guided surgery, Intraoperative imaging

## Abstract

**Background:**

Sacral neuromodulation operations have usually been performed based on 2D fluoro images. However, sacral nerve stimulation lead implantation may be challenging when the normal anatomy is confused by obesity or congenital anomalies. Thus the surgical navigation and intraoperative imaging methods could be helpful as those same methods have proven to be feasible methods for guiding other surgical operations. Our recent knowledge about the O-arm in trauma pelvic operations encouraged us to evaluate the usefulness of O-arm guided navigation in sacral neuromodulation. Similar navigation would be useful for complex sacral nerve stimulation lead implantations.

**Methods:**

In this preliminary article we report our experience of utilizing the orthopedically optimized O-arm to implant the S3 stimulation electrode in a patient. The 3D O-arm imaging was performed intraoperatively under surgical navigation control. General anesthesia was used. The obtained 3D image dataset was registered automatically into the patient’s anatomy. The stimulation needle was guided and the tined lead electrode was implanted using navigation.

**Results:**

The bony sacral structures were clearly visualized. Due to automatic registration, the navigation was practicable instantly after the O-arm scanning and operation could be performed successfully under navigation control.

**Conclusions:**

To our knowledge, this is the first published tined lead implantation which was guided based on the surgical navigation and intraoperative O-arm images. In this case, the applied method was useful and helped the surgeon to demarcate the region of surgical interest. The method is slightly more invasive than the formal technique but could be an option in anatomically challenging cases and reoperations. However, further evaluation with larger patient series is required before definitive recommendations can be made.

## Background

Sacral nerve stimulation (neuromodulation) has become a widely accepted method to treat various urological and gastrointestinal problems [1]. The technique is based on the stimulation of sacral nerves, usually S3, through the sacral foramina. Usually, the localization of the nerves is easy for an experienced surgeon. In some cases, however, problems can arise, especially with anomalous sacral anatomy or morbid obesity. Recently, complications associated with sacral neuromodulation for bowel problems were reported [2]. In a literature review, thirty-five incidences of problems associated with the implanted lead were detected, including 31 lead displacements/dislodgements/dislocations/migrations and 4 lead fractures [2]. Earlier, Siegel and colleagues [3] reported complications among the 914 test stimulation procedures done on the 581 patients, where 181 adverse events occurred in 166 of these procedures (18.2% of the 914 procedures). The vast majority of complications were related to lead migration (108 events, 11.8% of procedures) [3]. Technical advances, such as the introduction of the tined lead and the two-staged procedure, appear to have positively affected the adverse event and reoperation rates [4].

In neurosurgery, navigation has been used for about 20 years and the benefits are also obvious in some orthopedic operations [5]. An earlier publication describes the use of computed tomography for needle implantation guidance [6]. To evaluate the usefulness of surgical navigation in sacral neuromodulation, the O-arm was used for the implantation of a tined lead electrode in a patient already needing their third revision.

## Methods

### Patient

The patient is a 51 year-old female with multiple sclerosis. She has a neurogenic overactive bladder and severe constipation. In 2003 the patient underwent peripheral nerve evaluation (PNE). She saw no improvement in bladder symptoms but a clear alleviation of constipation, and underwent implantation using InterStim^R^. The tined lead technique and the left S3 were used, and the patient was satisfied with the operation. Overactive bladder symptoms were successfully treated with repeated botulinum toxin injections; the battery was changed in January 2007. The patient, however, did not have any feeling of stimulation and a revision was performed in December 2007. The left lying electrode was partially cut and a new tined lead electrode was placed on the right S3. During the following years, the patient was satisfied with stimulation but a high amount of current was needed. In winter 2011, the battery again was empty. Following X-ray it was noted that the electrode had moved outwards; therefore, a new electrode was placed on the right S4 in the second revision operation. The patient improved again. In autumn 2012, the patient again reported feeling no further stimulation and the measured impedances were over 4000 ohms. Because the patient was satisfied with the neuromodulation she wanted to have a revision. Due to the complicated history, already necessitating the third revision, the O-arm system was considered for the localization of foramina; the patient accepted the proposal and the operation was performed in January 2013. General anesthesia was used. Consent to publish her case was obtained from the patient. The treatment was conducted according to the Declaration of Helsinki.

### The O-arm

**Figure 1:**
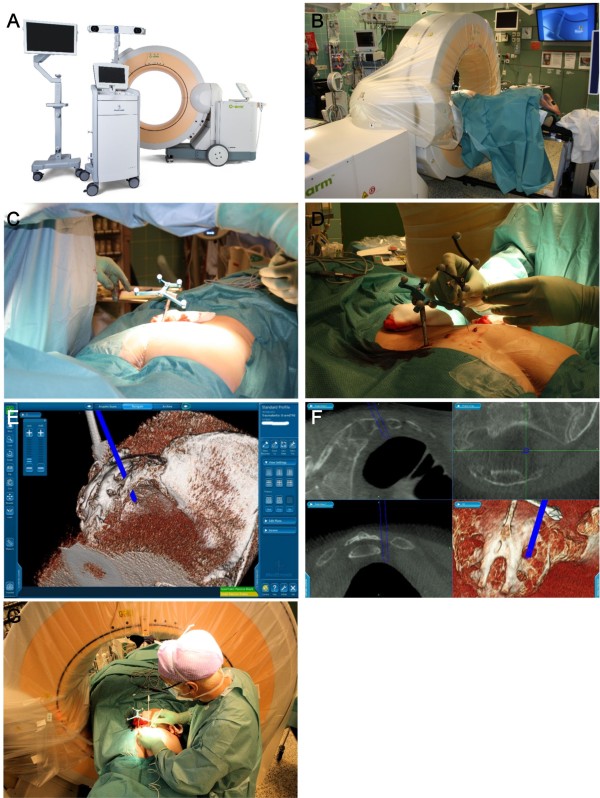
**Intraoperative figures of sacral nerve stimulator lead implantation.** The O-arm is a transportable CT-like scanner. With automated positioning, it can be easily placed around the patient on the radiolucent table to obtain 2D fluoroscopic images and 3D scanning in desired directions, and repeated when necessary **(A)**. The patient’s position can be adjusted optimally to achieve a surgically and anesthetically comfortable situation **(B)**. Patient tracker fixed to the bone **(C)**. The multipurpose SureTrack instrument tracker was fixed to the needle and used for navigation **(D)**. Needle inside S3, 3D image **(E)**. The sacral structures are clearly visible in the O-arm images; it was possible to guide the instruments used in the correct direction and to the right depths. Inserts with 2D and a 3D images **(F)**. Lead implantation; the O-arm has been moved in the cranial direction allowing free access to the operation area **(G)***.*

The O-arm (Medtronic Inc., Louisville, CO, USA) is a mobile 2D/3D X-ray imaging system optimized for bony structures in spinal and orthopedic surgery. Scanning is based on a flat panel detector and cone-beam technology producing 192 slices in 13 seconds in the standard mode. Pixel size is 0.415 × 0.415 mm within a slice thickness of 0.833 mm. The size of the scanned cylindrical 3D volume was 16 cm × 21 cm (length × diameter), which fulfilled the requirements for obtaining a full scan of the sacrum (Figure 1A).

### Surgical table

The patient lay prone on four-point cushions and on a carbon fiber operating table (Mizuho OSI, Modular Table System, Union City, CA, USA). The patient’s head was placed on the soft mask, and slid into the socket of the C-Flex Polar Head Positioner System (Allen Medical Systems, Acton, MA, USA), which was fixed to the carbon fiber rails (Figure 1B).

### Imaging

For the scanning, the patient and the O-arm’s gantry were positioned so that the sacrum was in the O-arm’s isocenter; this was verified using lateral and vertical 2D fluoroimages. The 3D data set were obtained utilizing the standard mode for the pelvic region with scanning values for M-size patient. Images were imported automatically to the surgical navigation system.

### Surgical navigation

The applied surgical navigation system was StealthStation S7 (Medtronic Inc., Louisville, CO, USA), which utilizes the optical localization method. The navigation software was spine software, which enables automated registration between the scanned images and patient anatomy. Automated registration requires a line of sight from the optical camera to the active O-arm tracker and the passive patient tracker when scanned. The patient tracker was fixed to the patient’s left posterior sacral crest using a percutaneous pin (Figure 1C).

### Procedure

After fixing the pin, the earlier placed and partially cut left S3 and right S4 electrodes were removed. It had been previously decided that right S3 was to be used. A foramen needle was fixed to the patient tracker (Figure 1D). Using the monitor made it very easy for the urologist to introduce the needle inside the right S3 (Figure 1E and F). During testing an anal contraction was confirmed and a tined lead was introduced into the foramen. During the operation, the O-arm did not prevent the surgeon from working in any way (Figure 1G). With a connection cable, the electrode was attached to the old InterStim^R^ pulse generator. The wounds were closed in layers and dressed. The recovery of the patient was uneventful and she was satisfied with the stimulation.

### Radiation exposure

During 2D and 3D scanning, the patient was exposed to ionizing radiation. The 2D fluoroimages were used to center the O-arm in the optimal position for 3D-scanning and verifying the final location of the implanted electrode. The 3D scan was required for the navigation. In the 2D mode, total exposure time was 13.51 sec and the radiation dose was comparable to those when using conventional C-arms. In the 3D mode, the CTDI (computed tomography dose index) was calculated as 14.08 mGy and the DLP (dose length product) was 225 mGycm. The Finnish Radiation and Nuclear Safety Authority (Säteilyturvakeskus, STUK) has not yet published the dose limits for the pelvic CTs. However, radiation doses are quite low compared to the acceptable dose limits in some other countries in Europe (CTDI: 14 – 35 mGy and DLP: 450 – 650 mGycm) [7]. Ionizing radiation doses should be adjusted using the collimation and limiting 2D area and by shortening the length of the 3D volume.

## Results

The use of the O-arm was fluent. This was possible with the automated positioning system which did not cover region of the surgical area. The sacrum and the foramina were clearly visualized. To achieve clear demarcation, the values of brightness and contrast had to be adjusted. Due to automatic registration, the navigation was practicable instantly after the O-arm scanning.

The locations of the skin incision and the trajectory of the needle could be determined based on the navigation data as the foramina were well demarcated. During the operation, the surgeon was able to control the movements of the needle and to find the correct depth for both the needle and the implanted electrode. The treatment was successful with ongoing very good treatment response to constipation in September 2013.

## Discussion

The technical advances of sacral nerve stimulation testing phase, namely the use of a tined lead and two phase testing, have improved the results during the last decade [4]. However, the complex anatomical situations may obliterate these advances and can result in increased complication rates and impaired testing phase results. In the search for safe techniques and to overcome challenging situations, the use of navigation for lead implantation during sacral nerve stimulation was evaluated and found to be successful. Navigation utilizing the O-arm has been shown to be useful in spinal trauma surgery, especially in pedicle screw positioning [8]. To our knowledge, this is the first report of surgery when navigation has been used in the implantation of the sacral nerve stimulation lead. In our clinic, we have performed neuromodulations since 1996, when the first test was performed; the first implantation was in September 1997. The Finnish urological patient material was published in 2011 [9]. One of the authors (PH) also has some experience of the treatment of fecal incontinence with neuromodulation and has performed implants in some myelomeningocele patients.

Using anatomical landmarks and fluoroscopy makes it less difficult to find the foramina [10]. The technique of sacral nerve stimulation has evolved during recent decades. At first, peripheral nerve evaluation (PNE) was performed using a very thin temporary electrode [11,12]; later, the use of a tined lead electrode became routine [13]. Sometimes, however, difficulties can arise in the localization of the foramina, especially if there are anatomical variations, like in myelomeningocele patients. We think that navigation using an O-arm could be beneficial in these cases. Lansen-Koch and associates [14] performed peripheral nerve evaluation for 10 patients with spina bifida: in two, PNE was not possible, 3/10 patients had more than 50% improvement and proceeded to a permanent implant, while in one patient it was not possible to perform the implantation. The authors performed an open operation to prevent damage to the nerves. However, using navigation, open surgery could probably be avoided.

Of course, the use of the tracker makes the procedure slightly more invasive. The implantation of the percutaneous pin, however, takes on only a few minutes and can be performed under local anesthesia. In overweight or obese people, however, it is often difficult to find the appropriate foramen. Our patient was relatively thin, so we cannot discuss how the technique would work in obese patients. However, as the bony structures are the landmarks for navigation, this technique should also be applicable with obese patients. Bending of the foramen needle will cause problems in navigation, meaning that a stiffer needle may be necessary.

## Conclusions

As far we know, this is the first published tined lead implantation which was guided based on the surgical navigation and intraoperative O-arm images. The method is slightly more invasive than the formal technique and much trickier and expensive than fluoroscopy. However, it could be an option in anatomically challenging cases and reoperations. Overall, there is a place for this technique, but further studies are needed.

### Consent

Written informed consent was obtained from the patient for publication of this Case report and any accompanying images. A copy of the written consent is available for review by the Editor of this journal.

## Competing interests

Pekka Hellström. Consultant: Glaxo Smith Kline, SCA Hygiene Products, PhotoCure, Pfizer, Allergan. Speaker Honorarium: Medtronic, Photocure, Allergan, Abbott, Pfizer. Trial participation: Glaxo Smith Kline, Amgen, Orexo, Novartis. Travel grants: Lilly, SCA Hygiene Products, PhotoCure, Allergan, Pfizer, Glaxo Smith Kline. Jani Katisko. Consultant: Medtronic. Pertti Finnilä. Equity interests: Medtronic. Other, employee: Medtronic. Markku Vaarala. Consultant: Amgen, Astellas, Janssen. Speaker Honorarium: Amgen, Astellas, Novartis. Trial participation: Amgen, Astellas, AstraZeneca, Pfizer, Orexo, Millenium Pharmaceuticals, Janssen.

## Authors’ contributions

PAH, JK and PF participated in the design of the study. PAH performed the operation described. JK and PF coordinated the operation including the use of the O-arm and helped to draft the manuscript. PAH and MHV drafted the manuscript. All authors read and approved the final manuscript.

## Pre-publication history

The pre-publication history for this paper can be accessed here:

http://www.biomedcentral.com/1471-2490/13/48/prepub
